# St John’s wort (*Hypericum perforatum*)-induced psychosis: a case report

**DOI:** 10.1186/s13256-017-1302-7

**Published:** 2017-05-15

**Authors:** Maria Ferrara, Francesco Mungai, Fabrizio Starace

**Affiliations:** 1 Dipartimento di Salute Mentale e Dipendenze Patologiche, Modena, Italy; 2 Department of Mental Health & Drug Abuse, AUSL Modena, Viale Muratori 201, 41124 Modena, Italy

**Keywords:** *Hypericum*, St John’s wort, Mental health, Psychosis, Complementary and alternative medicines, Herbal remedies

## Abstract

**Background:**

St John’s wort (*Hypericum perforatum*) has been known for centuries for its therapeutic properties and its efficacy as an antidepressant has been confirmed by a growing body of evidence. During the last two decades it has also come to prominence with a wider public, due to advertising efforts across Europe and United States of America. However, its availability without prescription, as an over-the-counter medication, raises some concern regarding its clinical management and unsupervised administration to individuals with psychopathological risks. To date, the evidence available regarding the administration of *Hypericum* in people with severe mental health problems is still meager and refers mainly to affective disorder spectrum or psychotic relapse in people with established diagnoses. To the best of our knowledge, this is the first report regarding the onset of psychotic features in a patient presenting with psychotic diathesis.

**Case presentation:**

The case discussed in this report is a 25-year-old white man, not known to the psychiatric services, with a history of brief and self-remitting drug-induced psychosis and a positive family history of psychotic depression. He was admitted to hospital due to the onset of florid psychotic symptoms concomitant with self-administration of *Hypericum perforatum*.

**Conclusions:**

The aim of this report is to promote further systematic research, draw the attention of clinicians to the potential risks of *Hypericum* precipitating psychosis, and raise awareness among health professionals to investigate and caution their patients on the haphazard use of phytotherapeutics such as *Hypericum*.

## Background

*Hypericum perforatum* (St John’s wort) has been known for its therapeutic properties, such as anti-inflammatory, antiseptic, and antidepressant, since the times of ancient Greece and interest in this plant has been documented throughout history [1]. Over the last two decades, St John’s wort has gained significant relevance in the field of psychiatry for the treatment of mild-to-moderate depression. St John’s wort has been investigated, providing evidence of its efficacy and tolerability profile [2–7], and it has been included within internationally acknowledged guidelines [8]. At the same time, it has been increasingly advertised and commercialized across Europe and the United States of America as an over-the-counter medication. However, its rising popularity could also mean an increased risk for people self-medicating for potentially severe psychopathological conditions, for which there is no evidence of efficacy and tolerability, without receiving any professional supervision. In fact, even though a recent meta-analysis has provided further evidence supporting the comparability of St John’s wort to standard selective serotonin reuptake inhibitors (SSRIs), with regards to mild and moderate depression [7], to date, still too little is known about its safety, recommended dosage, and side effects in people at risk of psychiatric disorders.

St John’s wort contains a combination of different components, which makes the interpretation of clinical trials rather complex [9]. However, among its active constituent fractions (for example, hypericin, hyperforin, and polyphenols) consensus has now been reached regarding the key role of the hyperforin component in antidepressant activity [7]. Furthermore, in many cases, the formulations available and advertised across countries as over-the-counter remedies for the treatment of mood disorder, anxiety, and jet lag syndrome, consist of a combination of *Hypericum* and other phytotherapeutics (for example, valerian) or melatonin.

St John’s wort is a complex mixture containing a variety of constituents; some of these components are potent enzyme inducers and their pharmacokinetic interactions should be particularly cautioned with concomitant drug use [10, 11]. In particular, hypericin and hyperforin are reported to be respectively CYP1A2 and CYP3A4 inducers [7]. Thus, attention should be paid when *Hypericum* is administered in combination with other medications in general [12, 13] and more specifically with drugs such as antiretrovirals [14], cyclosporine [15], anticoagulants [16], hormonal contraceptives [17], and other psychotropic agents such as antidepressants [15] and antipsychotics [18]. The number of neurotransmitter systems potentially involved in the mechanism of action of *Hypericum* is remarkable, encompassing not only monoamines (serotonin, noradrenaline, and dopamine), for which the antidepressant effect is hypothesized, but also glutamate and ion channels [19, 20].

To the best of our knowledge, to date, the evidence available regarding the risk posed by *Hypericum* in terms of psychiatric adverse effects, with reference to its potential to precipitate psychosis, is limited [21]; the evidence is confined essentially to some case reports describing the onset of manic symptoms [22–25], two cases of psychotic relapse [26], and a case report regarding the onset of a first episode of psychosis in a 39-year-old Japanese woman who self-medicated with a high dosage of *Hypericum* during a spell of mild depressed mood [27].

## Case presentation

The case discussed refers to a 25-year-old white man, previously unknown to the psychiatric service, seen at Accident and Emergency (A&E) by the psychiatrist on call and immediately admitted to our acute psychiatric unit due to his florid psychotic symptoms. He was accompanied by two friends who described him as having been “off and strange” over the last few days, reporting that it looked like he was under the effect of some sort of illicit drug. His clinical picture was characterized by disorganized speech, paranoid thinking, and delusions of influence, such as thought control and beliefs that his mind was being read. He also presented with pervasive somatoform preoccupations regarding his internal organs “being displaced” and a form of Capgras delusion towards his parents. He denied experiencing auditory hallucinations. On the ward he remained very quiet, although no objective mood disturbances were detected. Nevertheless, he complained of weakness and to be struggling with a “period of distress”; he could not elaborate further. He did not present anxiety or sleep disturbances. His blood test results were within the normal range and he did not show any neurological abnormalities. The result of his toxicological blood screening was negative. He was initially administered risperidone (9 mg daily), subsequently switched to paliperidone (6 mg daily) due to the onset of extrapyramidal symptoms and a better tolerability profile. His condition settled fairly quickly and, due to a substantial improvement in his clinical picture, after 15 days of hospital stay he was discharged with a diagnosis of schizophreniform disorder. Due to poor insight and his reluctance to continue taking the medication, he was started on the long-acting antipsychotic, Xeplion (paliperidone palmitate), 100 mg injection monthly.

Over the following 3 months he attended follow-up visits at the local community mental health service. His clinical picture remained stable and his insight, energy, and global functioning gradually improved. However, during the follow-up visits he gave an account of a previous psychotic episode, 9 months before the index episode, concomitant with cannabis abuse. He reported having seen a specialist and being offered olanzapine 2.5 mg daily, which he declined along with the follow-up visits. He claimed that since then he had stopped taking illicit drugs. Subsequently, he reported an improvement in his mental state. However, 3 months prior to the admission to our psychiatric ward, he started experiencing weakness, exhaustion, and severe stomach discomfort. He reduced his food intake, losing up to 8 kg, and started feeling so weary he decided to resign from his job. He resolved to see his general practitioner, who arranged to carry out some investigations. An esophagogastroduodenoscopy (EGD) showed the presence of multiple stomach erosions and *Helicobacter pylori* infection which could explain his stomach pain and physical problems. Nevertheless, he turned down the treatment offered by his general practitioner, due to his personal inclination against pharmaceutical drugs, and started to self-medicate with *Hypericum*. The formulation taken was herbal aqueous infusion (sachets of the herb brewed in water), which has been reported as a rich source of *Hypericum* components (that is, hypericins and flavonoids), comparable with tablets and capsules; he took doses recommended for mild/moderate depressive episodes [28]. He recounted a dose of 4 g of herbal mono-preparation per infusion, and quantified his average intake as four cups daily. He admitted to have continued taking *Hypericum* nonstop until he was admitted to our acute psychiatric unit and it was during that time that he could recall the exacerbation of psychotic symptoms. Later, he also informed the clinicians that his father had experienced psychotic depression, of which he was not aware at the time of admission.

## Discussion

The lack of detailed information on the composition of the St John’s wort tea mono-preparation taken by our patient and suspected to have caused the described event should be acknowledged as a limitation. Moreover, it is not possible to establish a definitive causal link regarding the onset of a psychotic episode precipitated by self-administration of *Hypericum*. However, we can hypothesize that in this young and vulnerable individual, with a known genetic predisposition and a previous drug-induced psychotic episode, the unsupervised self-administration of *Hypericum* could have played a determinant role in the onset of the psychopathological conditions leading to his urgent hospital admission. In addition, this case suggests that treatment with antipsychotics may be effective for *Hypericum*-associated psychosis.

## Conclusions

To date, the evidence available regarding the safety profile of *Hypericum* in people with risk factors for psychosis is still insufficient and more systematic research is necessary; clinicians and health professionals in general should consider the risk of *Hypericum* precipitating psychosis. Moreover, since patients generally do not volunteer information about usage of alternative medicines, it is particularly important that physicians actively elicit herbal remedy use in their history taking, particularly in cases of sudden-onset of psychosis. Finally, cautioning patients on the haphazard use of herbal remedies, such as *Hypericum*, should be part of best practice in health care.

## Abbreviations

A&E: Accident and Emergency
EGD: Esophagogastroduodenoscopy
SSRI: Standard selective serotonin reuptake inhibitor
